# Exploring Ag(111) Substrate for Epitaxially Growing Monolayer Stanene: A First-Principles Study

**DOI:** 10.1038/srep29107

**Published:** 2016-07-04

**Authors:** Junfeng Gao, Gang Zhang, Yong-Wei Zhang

**Affiliations:** 1 Institute of High Performance Computing, A*STAR, 138632, Singapore

## Abstract

Stanene, a two-dimensional topological insulator composed of Sn atoms in a hexagonal lattice, is a promising contender to Si in nanoelectronics. Currently it is still a significant challenge to achieve large-area, high-quality monolayer stanene. We explore the potential of Ag(111) surface as an ideal substrate for the epitaxial growth of monolayer stanene. Using first-principles calculations, we study the stability of the structure of stanene in different epitaxial relations with respect to Ag(111) surface, and also the diffusion behavior of Sn adatom on Ag(111) surface. Our study reveals that: (1) the hexagonal structure of stanene monolayer is well reserved on Ag(111) surface; (2) the height of epitaxial stanene monolayer is comparable to the step height of the substrate, enabling the growth to cross the surface step and achieve a large-area stanene; (3) the perfect lattice structure of free-standing stanene can be achieved once the epitaxial stanene monolayer is detached from Ag(111) surface; and finally (4) the diffusion barrier of Sn adatom on Ag(111) surface is found to be only 0.041 eV, allowing the epitaxial growth of stanene monolayer even at low temperatures. Our above revelations strongly suggest that Ag(111) surface is an ideal candidate for growing large-area, high-quality monolayer stanene.

Quantum spin Hall (QSH) insulators are new states of condensed matter, in which insulating bulk and metallic edge states coexist^1^,^2^. Since the electrical conduction along their edges is dissipationless, they are promising for the realization of novel devices with minimum energy dissipation. In general, the working temperature of QSH insulators depends on the gap of bulk state. Thus the search for QSH insulators with large gap has drawn a cornucopia of attentions^3^,^4^,^5^,^6^,^7^,^8^,^9^,^10^,^11^,^12^,^13^,^14^,^15^. Recently, stanene, a monolayer of tin film, has attracted extensive interest due to its sizeable bulk gap^16^,^17^,^18^,^19^,^20^,^21^,^22^. Interestingly, the strong spin-orbital coupling (SOC) in stanene is able to open a 73.5 meV nontrivial band gap, which is significantly larger than the slight gap of graphene, 1.55 meV gap of silicene and 23.9 meV gap of germanene^20^. It is worth noting that surface halogenation is able to further enlarge the bang gap of stanene to more than 300 meV^16^,^17^,^19^. Such a large band gap instigated by SOC effect is sufficient for application as a room-temperature QSH insulator. Besides, the Fermi velocity (υ_F_) of its helical edge state is up to 6.8 × 10^5^ m/s and 7.2 × 10^5^ m/s for fluorinated and chlorinated stanene, respectively. Therefore, stanene and its derivatives exhibit both QSH temperature and Fermi velocity superiorities over the well-established HgTe quantum well, in which the QSH effect exists only below 10 K and υ_F_ is ~5.5 × 10^5^ m/s^16^.

Stanene features both the honeycomb lattice structure and the Dirac cone electronic structure^20^,^23^. Unlike graphene, stanene has a much weaker π-π bonding. As a result, a low-buckling arising from σ-π hybridization is formed to stabilize its two-dimensional (2D) lattice structure, a common phenomenon in group-IV 2D monolayers^24^,^25^ beyond graphene. Not surprisingly, many of its fascinating electronic properties originate from this unique structure.

Multilayer stanene films or α-Sn thin films were grown epitaxially on InSb^21^,^26^ and CdTe^27^ using molecular beam epitaxy (MBE). Only recently, monolayer and few-layer stanene were successfully grown epitaxially on Bi_2_Te_3_ (111) surface via MBE and the obtained atomic structures and their electronic properties were studied by using scanning tunneling microscopy (STM), angle-resolved photoemission spectroscopy (ARPES) and first-principles calculations^28^. It was found that the stanene epitaxially grown on Bi_2_Te_3_(111) was a mixture of monolayer, bilayer and multilayer^28^. The underlying reason for forming such a mixture may be that the sharp steps on Bi_2_Te_3_(111) surface, which are ~1 nm in height, are able to block the continuous growth of stanene by suppressing the growth fronts to cross the “uphill” steps^28^. Evidently, substrate plays a critical role in the growth of stanene monolayer.

Ag (111) surface is a commonly-adopted substrate for the growth of group-IV monolayers. For example, large-size monolayer silicene structures with well-ordered patterns^29^, 3 × 3^30^, 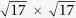
31 and 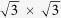
32,33 were observed experimentally on Ag (111) surface^34^. Very recently, a series of monolayer boron sheets (borophene) were successfully synthesized on Ag(111) surface via MBE, further demonstrating its versatility^35^,^36^. In general, as an ideal substrate for epitaxial growth, a low and uniform diffusion barrier for adatom is expected to speed up the growth process. Moreover, a moderate interaction strength between the substrate and the 2D monolayer is required: It should be strong enough to support the 2D monolayer but should not be so strong to destroy its crystal structure. Previous studies clearly showed that Ag(111) surface is such an ideal substrate for the epitaxial growth of silicene^29^,^30^,^31^,^32^,^33^,^34^,^35^,^36^,^37^. However, it is still an open question whether monolayer stanene can be grown on Ag(111) surface. Specifically, we need to answer the following questions: What is the nature of the interaction between stanene epilayer and Ag(111) surface? What is the resulting epilayer structure of stanene upon such interaction? If the epilayer is detached by etching away Ag substrate, can the epilayer fully recover to the free-standing lattice structure of monolayer stanene? Clearly, answers to these questions are not only of scientific interest, but also of significant consequence on whether Ag(111) is suitable for the epitaxial growth of large-area, high-quality stanene monolayer.

In this paper, we examine the interaction between monolayer stanene and Ag(111) surface via first-principles calculations. By varying their epitaxial relation, we find that different epilayer stanene structures can be formed, and surprisingly, all the epilayer structures retain the hexagonal lattice of stanene. The average distance between all the stanene configurations and Ag (111) surface is found to be in the range of (2.41~2.48 Å), suggesting that monolayer stanene and Ag(111) interact chemically. Since this average distance is comparable to the step height (2.4 Å) on Ag (111) surface, it is expected that stanene flakes are able to continuously grow “uphill” and cross over the surface steps. Remarkably, we find that all the epilayer structures are able to fully recover the free-standing lattice structure once the Ag substrate is chemically etched away. Moreover, the diffusion barrier of Sn adatom on Ag(111) is found not only low (0.04 eV) but also nearly uniform, indicating that the epitaxial growth could be conducted at low temperature. Our studies here provide compelling evidences that Ag(111) surface is an ideal candidate for growing monolayer stanene.

## Results

**Figure 1:**
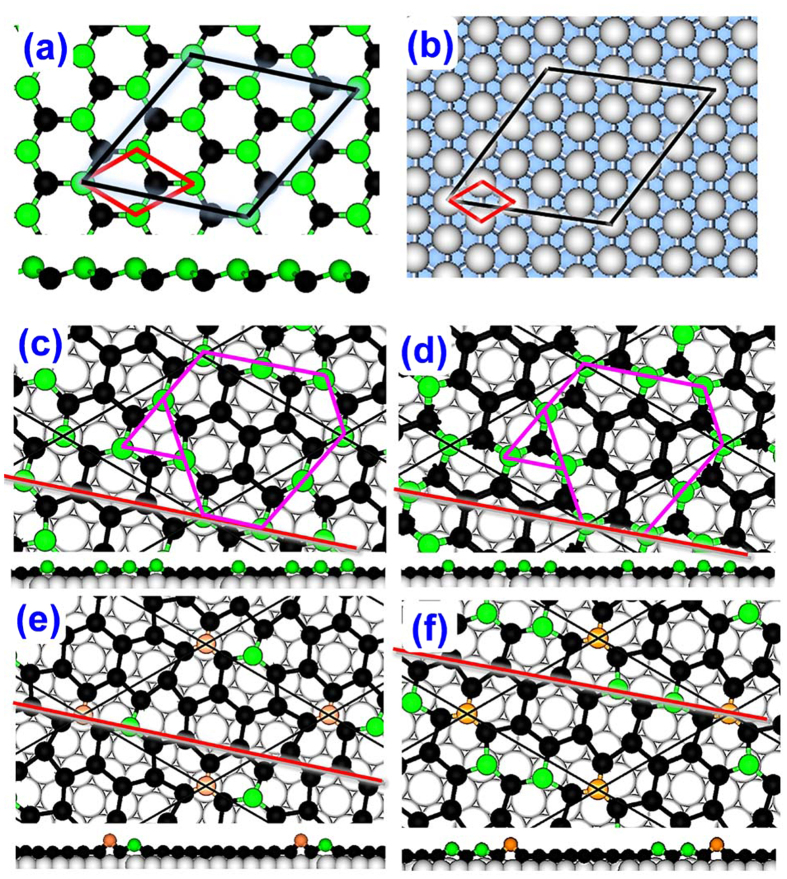
The top view and side view of free-standing monolayer stanene (**a**) and Ag(111) lattice (**b**), black lines represent the co-periodic supercell of 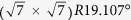 stanene and 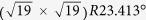 Ag(111) surface, and red lines represents the primitive cells, respectively. The top view and side view of four optimized stanene/Ag(111) superstructures: Structures S1 (**c**), S2 (**d**), S3 (**e**), S4 (**f**). Here purple lines represent the unique patterns of stanene in structures S1 and S2 (**c**,**d**), and red lines indicate a typical zigzag direction. The orange, green and black balls represent the Sn atoms on different layers, and grey balls represent the Ag atoms in the topmost layer of Ag(111) surface.

Although we have constructed a series of initial configurations of stanene with respect to Ag(111) surface, only four stanene epilayer structures as shown in Fig. 1(c–f), which are sequentially named as S1 to S4, are observed after the structural relaxation, indicating that some of the initial configurations share the same final epilayer structures. Most importantly, we find that all the considered structures of stanene monolayer retain the hexagonal lattice structure upon overlying on Ag (111) surface after energy optimization. Also these epilayer structures share a similar binding energy and a similar average height with the substrate as shown in Table 1. Although the hexagonal lattice structure is retained, their buckling patterns are different among themselves and also different from that of the free-standing stanene monolayer. From the side views of these structures, it is seen that the Sn atoms in S1 and S2 fall roughly into two layers (atoms in green and black, respectively). The two-layer structure of S1 and S2 is very similar to that of free-standing stanene except that the ratio of the number of atoms in the upper layer and bottom layer is different, that is, 28.6% in S1 and S2 vs. 50% in free-standing stanene. Although the epilayer structures of S1 and S2 are nearly the same, their buckling arrangements and the related positions on Ag(111) are different: The vertex Sn atoms of S1 are right on top of Ag atoms, while the vertex Sn atoms of S2 are at top of hollow sites. In addition, for S2, there is a flat black hexagon surrounded by the upper Sn atoms (colored in green), but for S1, there is no such flat Sn hexagon. As a result, the average distance of Sn atoms in S1 (2.408 Å) is slightly lower than that in S2 (2.411 Å) (see Table 1).

From Table 1, it is note that S3 possesses the highest binding energy of −0.059 eVÅ^−2^, implying the motif in S3 is energetically superior to other structure, which waiting the experimental verification in the future. Compared to S1 and S2, apparently, S3 and S4 possess different buckling patterns as shown in the side views of Fig.1(e,f). Different from the obvious two-layer characteristic in S1 and S2, one Sn atom in the supercell is distinctively higher than others (marked in orange in Fig. 1(e,f)). In S3, this topmost Sn atom is right on top of an Ag atom, while in S4, it takes the hollow site. Although the local buckling heights in S3 and S4 increase, the hexagonal lattice structures are still preserved, as shown in Fig. 1(e,f). For S4, both its binding energy (−0.058 eVÅ^−2^) and its average height (2.444 Å) are in the middle of the four configurations.

Remarkably, the heights of all the four stanene configurations are comparable to the step height (~2.40 Å) of Ag(111) surface observed experimentally^32^,^38^. In general, a group-IV 2D material interacts with a flat metal surface primarily through its hybridized out-of-plane π orbitals and metal *d* bands. However, this picture may break down when the growth front of the monolayer material encounters a step on the substrate surface. In particular, if the height of the 2D monolayer is significantly lower than the step height of the metal surface, strong σ-like bonds may form between the 2D monolayer and metal substrate, which may suppress the “uphill” step crossing. Conversely, if the height of 2D monolayer is larger than the step height of the substrate surface, the σ-like bonds at the step edges are unlikely to form, allowing the 2D monolayer to grow over steps. This effect has been observed in the growth of graphene^39^,^40^ and silicene^32^ on various metal substrates. Hence, we can deduce that a stanene flake is expected to grow across the surface steps on Ag(111) surface.

**Figure 2:**
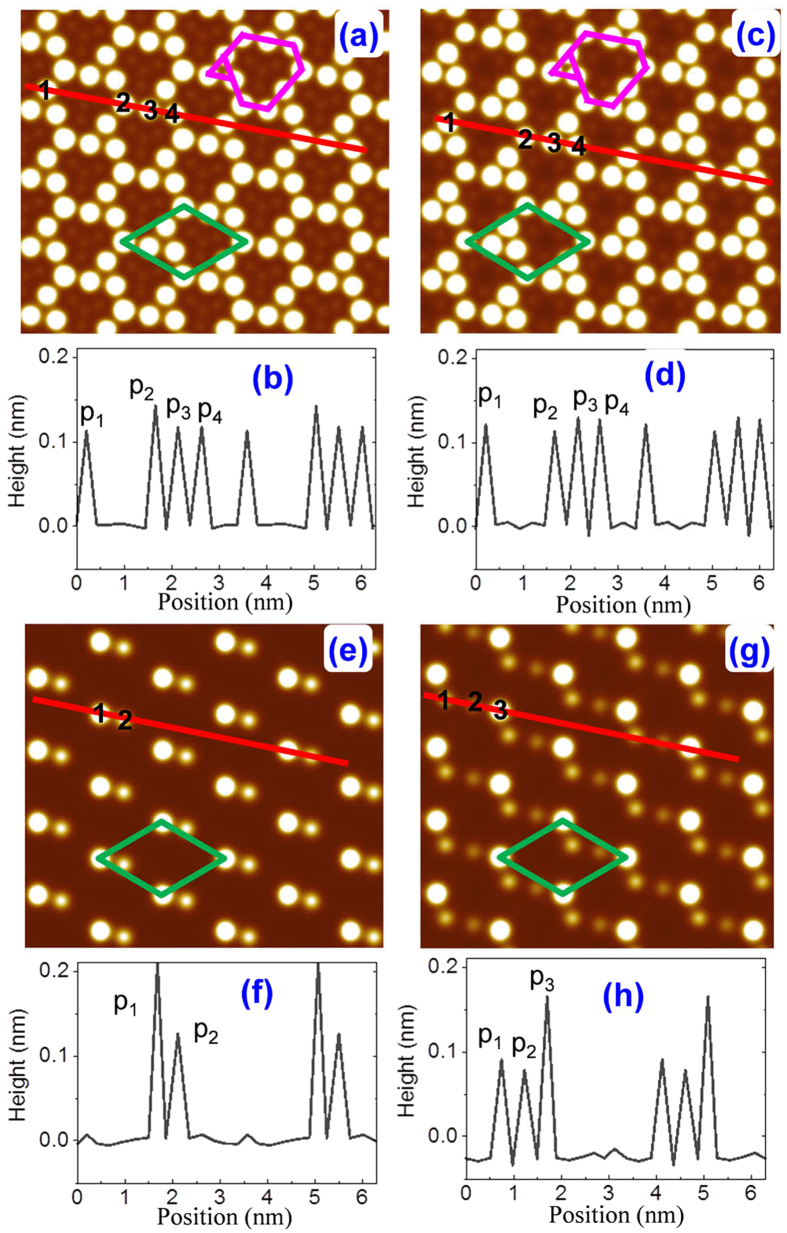
The simulated STM images of stanene reconstructions on Ag(111) surface at a bias of +1V, and the related height line profiles along the red lines: Structures S1(a,b), S2(c,d), S3(e,f), S4 (g,h).

To facilitate the experimental identification of these four structures, we simulate their STM images^41^ and plotted the height line profiles along the zigzag direction of stanene in Fig. 2. A comparison of Figs 2 and 1 shows that only these topmost Sn atoms are visible in STM imaging due to the buckled configuration. The STM images of structure S1 (Fig. 2(a)) and structure S2 (Fig. 2(c)) share similar patterns, both exhibiting a network of bright triangles and hexagons. Only small structural differences can be observed from the height line profiles as shown in Fig. 2(b,d): The peak P_2_ is the highest peak in S1 (0.142 nm) while it is the lowest peak in S2 (0.112 nm), and the peaks spacing *d*_*23*_ (*d*_*34*_) is 0.474 (0.500) nm in S1 but it is 0.499 (0.456) nm in S2. These features are consistent with their different positions: P2 atom in S1 is right on top of an Ag atom of Ag(111) surface, while in S2 configuration, it is on top of a hollow site of Ag(111). The density of bright spots in S3 STM image (Fig. 2(e)) is much lower than those of S1 and S2. There is one sharp bright spot per supercell, corresponding to the topmost atom (Fig. 1e) in the height line profile (Fig. 2(f)). Near the strong bright spot, however, there is a dim spot, which corresponds to the second highest peak in Fig. 2(f). The distance between the two spots is about 0.432 nm. The STM image of S4 [Fig. 2(g)] is similar to that of S3, except that there is an extra dimmer spot. The buckling heights of S4 are 0.090 nm, 0.077 nm, 0.165 nm, and their separations are 0.487 nm and 0.483 nm, respectively.

It should be noted that the overall buckling heights in S1 and S2 are about 0.1 nm, very similar to that of free-standing stanene. In addition, the STM images of S1 and S2 possess C_3_ symmetry, nearly resembling the C_3v_ symmetry of freestanding stanene. Moreover, although the local buckling height of S3 is more than twice of that of free-standing stanene, the epilayer structure still keeps its structural integrity on Ag(111) surface. The average binding energy (see Table 1) of the epilayer stanene on Ag(111) surface is from −0.057 eVÅ^−2^ to −0.059 eVÅ^−2^. This value is about 2~3 times of that of graphene on Cu(111) and Ni(111) surfaces^42^, and is comparable with that of silicene on Ag(111) surface, which is about −0.056 eVÅ^−2 ^34,38. Similar to silicene, the interaction between Sn atoms and Ag(111) surface is chemical in nature. Importantly, upon their chemical interaction, all the epilayer lattice structures of stanene still preserve its hexagonal lattice structure.

**Figure 3:**
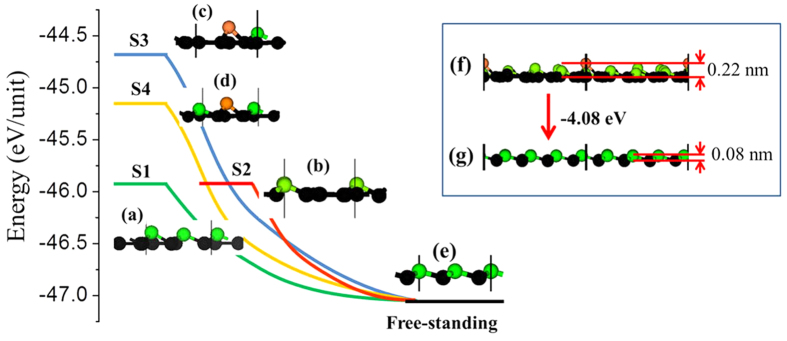
The self-recovery and the related energy variations of the stanene structures after desorption from Ag(111) surface: four representative structures S1 (**a**) and S2 (**b**), S3(**c**), S4(**d**); the low-buckling free-standing stanene (**e**). Another self-recovering process of (4 × 4) stanene detaching from the 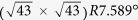 Ag(111) supercell: the initial distortion of (4 × 4) stanene (**f**) and the low-bucking free-standing stanene after the spontaneous transformation (**g**).

In a recent experimental study on the growth of silicene on an ultra-thin silver film^43^, a free-standing silicene was successfully obtained by etching away the Ag film without destroying silicene. With the obtained silicene sample, a silicene-based field effect transistor (FET) at room temperature was demonstrated^43^. Then, a question naturally arises: What will happen to the four representative stanene epilayer structures if they are detached from Ag(111) surface by etching away the Ag substrate? To answer this question, we optimize the four epilayer stanene structures by removing the Ag substrate. The changes in the total energy with respect to different structures in the desorption process are shown in Fig. 3. These energy changes are related to their local buckling heights in the epilayer structures. S1 and S2 have a similar low buckling pattern, and thereby, their intrinsic energies are also similar (see Fig. 3(a,b)), which are about 1.25 eV lower than that of S3, the highest buckling structure (Fig. 3(c)), and 0.78 eV lower than that of S4, the second highest buckling structure (Fig. 3(d)). But after the structural relaxation, these four epilayer structures spontaneously transform into the same low-buckling structure, which is just the most stable monolayer stanene sheet. The large energy drops provide strong driving forces for the structure changes: ~1.13 eV for S1 and S2, 1.90 eV for S4 and 2.38 eV for S3, respectively. Hence, all the four structures are able to spontaneously recover to the perfect free-standing structure, regardless of their different epilayer structures on Ag(111) surface.

To further verify the structural integrity of stanene on Ag(111) surface and the spontaneous recovery of the free-standing stanene lattice structure after the detachment, we have constructed a large stanene/Ag(111) supercell, i.e. (4 × 4)*R0*°stanene on a 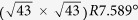 Ag(111) supercell (see Supplementary Information Fig. S1). It is found that the honeycomb structure of stanene is also preserved after structural relaxation. Compared with the four structures shown in Fig. 1, the bucking pattern of this large supercell can be recognized as a mixture of S1 (blue circle in Fig. S1), S2 (red circle in Fig. S1) and S3 (purple circle in Fig. S1). The binding energy between this epilayer stanene structure and Ag (111) surface is −0.058 eVÅ^−2^, and the height of the low-layer Sn atoms (black balls) to the Ag(111) surface is about 2.419 Å. Hence, both the binding energy *E*_*b*_ and the average height 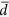 are between those of S1(S2) and S3. After the detachment, the initial epilayer lattice structure (Fig. 3f) also spontaneously transform into the prefect free-standing structure (Fig. 3g). During the self-recovery process, the energy drop is about 4.08 eV per unit. This large supercell calculation further confirms that stanene is able to keep its honeycomb structure on Ag(111) and self-recover to the perfect free-standing structure after the detachment.

**Figure 4:**
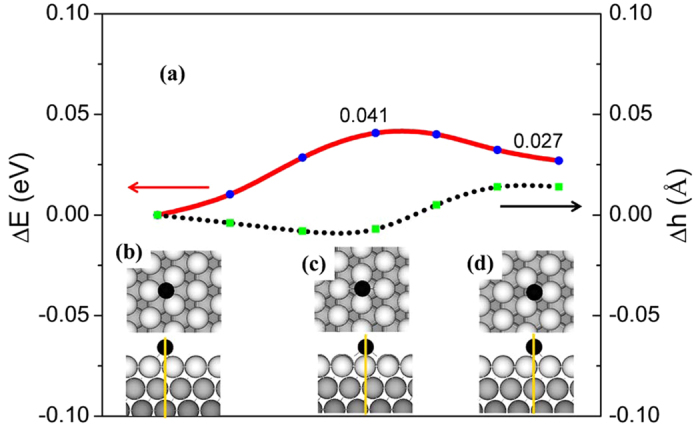
Energy profiles (red) and height variation (black dash) of Sn adatom diffusion along a representative path on Ag(111) surface. Both the energy and height of Sn adatom at the FCC position are set to zero. (**b**–**d**) top view and side view of the Sn diffusion pathway.

Our above results have demonstrated that upon chemical absorption on Ag(111) surface, monolayer stanene can form various epilayer structures. But all the epilayer structures are able to retain its hexagonal lattice structure. After being detached from the substrate, all the epilayer structures are able to spontaneously recover to the perfect free-standing monolayer stanene structure. Next we explore the diffusion of Sn atom on Ag(111) surface, which is crucial in the epitaxial growth of monolayer stanene from adatoms. Considering the symmetry of the primitive cell as shown in the insets of Fig. 4, we select a representative diffusive pathway on Ag(111) surface, and then calculate the energy profile of Sn adatom diffusing along this path by cNEB method^44^. It is found that the diffusion barrier (Δ*E*) is only 0.041 eV, similar to that of diffusion of Si atom (0.031 eV) on Ag(111)^34^, but much lower than that of diffusion of C atom on Cu surface (0.45 eV). The diffusion coefficient (*D*) of Sn atom on Ag(111) surface can be estimated by the formula:


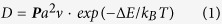


where ***P*** = 1/3 comes from the fact that there are three nearest probable positions to diffuse, ***a*** = 1.7 Å is the distance between a hollow site to its adjacent hollow site of Ag(111) surface, *v* is the atomic vibration frequency in the order of 10^13^ Hz, ***k***_***B***_ is the Boltzmann constant and ***T*** is the temperature. The diffusion coefficient is found to be 2 × 10^−4^ cm^2^s^−1^ (2 × 10^10^ nm^2^s^−1^) at 300 K, indicating that Sn adatom is able to diffuse easily and the growth of stanene on Ag(111) surface can be conducted at low temperatures. In addition to the low diffusion barrier, the change in the height (Δh) for Sn atom to diffuse from the FCC position to the bridge position and then to the HCP position on Ag(111) surface is very small, less than 0.05 Å. Here, it should be noted that both the energy and height of Sn atom at the FCC position are set to zero. Thus, the interaction strength between Sn adatom and Ag(111) surface is nearly homogeneous. For a 2D monolayer supported by a metal surface, a strong inhomogeneous interaction can potentially destroy its 2D lattice structure^34^. Therefore, the homogeneous interaction between Sn adatom and Ag(111) surface indicates that Ag(111) surface is an ideal candidate to grow monolayer stanene.

## Discussion

In summary, using first-principles calculations, we studied the interaction between monolayer stanene and Ag(111) surface. Depending on the initial epitaxial relations, various epilayer structures can be formed. Importantly, all the epilayer structures retain their honeycomb lattice structure, although exhibiting different buckling patterns. The average heights of these epilayer structures are all comparable to the silver surface step height, allowing easy crossing over the surface steps. In addition, the diffusion barrier of Sn atom on Ag(111) surface is not only low but also uniform, enabling the epitaxial growth of large-scale monolayer stanene on Ag(111) surface at low temperature. In addition, the simulated STM images as well as their height line profiles obtained here provide important references for future experiments. Most importantly, despite of the different epilayer structures formed on Ag(111) surface, all of them are able to spontaneously recover to the perfect free-standing stanene structure once they are chemically detached from Ag(111) surface. The present study strongly suggests that Ag(111) is an ideal substrate for epitaxially growing large-area, high-quality monolayer stanene. It is worth mentioning that Au^45^, Cu^46^, Pt^47^, and Ir^48^ surfaces share some similar features with Ag, and have been used to grow other 2D buckled films, such as borophene, silicene, germanene. Hence, these metal substrates may also be considered as potential candidates for the growth of stanene, which certainly deserves further study in the future.

## Methods

First-principles calculations are performed by the Vienna *ab initio* simulation package (VASP)^49^,^50^. The Perdew-Burke-Ernzerhof (PBE)^51^ generalized-gradient approximation (GGA) and projected augmented wave (PAW) method^52^ are used to treat the exchange-correlation functional and core electrons, respectively. The kinetic energy cutoff is 300 eV and the force criterion for structure optimization and climbing image nudged elastic band (cNEB)^44^ calculation is 0.02 eV/Å. The optimized lattice constant for the stanene and Ag(111) primitive cell is 4.676 Å and 2.932 Å, respectively. Therefore, we choose 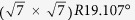
stanene (with a rotation of 19.107^o^ to the primitive cell) and 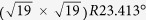 Ag(111) with three layers to build the co-lattice supercell [see Fig. 1(a,b)] with a large vacuum layer of 25 Å. Previous theoretical calculations have shown that a metal substrate with three atomic layers is sufficient to describe the structure of the supported 2D material samples, and the predicted results are in good agreement with experimental observations^34^,^53^,^54^,^55^,^56^. The stanene layer is stretched slightly to match the Ag(111) surface. In order to search different epilayer configurations of stanene monolayer on Ag(111) surface, several initial lattice configurations of stanene with respect to the Ag(111) surface are taken by moving the stanene layer along the 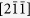 direction on the Ag(111) surface. The bottom Ag atoms are fixed and other Ag and Sn atoms are fully relaxed using a (2 × 2 × 1) k-mesh, which give good energy convergence for structural relaxations (See Fig. S2). Subsequently, a denser (6 × 6 × 1) k-mesh is used for energy calculations and STM simulations^41^.

## Additional Information

**How to cite this article**: Gao, J. *et al*. Exploring Ag(111) Substrate for Epitaxially Growing Monolayer Stanene: A First-Principles Study. *Sci. Rep.* **6**, 29107; doi: 10.1038/srep29107 (2016).

## Supplementary Material

Supplementary Information

## Figures and Tables

**Table 1:** The binding energy (E_b_) between the epilayer stanene and Ag(111) surface, which is calculated by using 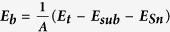, where E_t_, E_sub_ and E_Sn_ are the total energy of stanene/Ag(111) system, the energy of 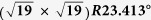 supercell of Ag(111) surface and the energy of 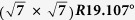 supercell of stanene, respectively.

	S1	S2	S3	S4
E_b_ (eVÅ^−2^)	−0.057	−0.057	−0.059	−0.058
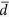 (Å)	2.408	2.411	2.484	2.444

The minus sign of E_b_ indicates that it is energetically favorable to have stanene absorbed on the Ag (111) surface. The average distance of the bottom stanene atoms to the Ag(111) surface is calculated by using 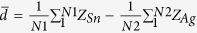, where N1 and N2 are the number of bottom stanene atoms (black balls in Fig. 1) and Ag atoms in the first layer of Ag(111), and Z_Sn_ and Z_Ag_ are the heights of Sn and Ag atoms, respectively.
