# Supplementary material for: Exploring Ag(111) Substrate for Epitaxially Growing Monolayer Stanene: A First-Principles Study
Source: Sci Rep. 2016 Jul 4;6:29107. doi: 10.1038/srep29107 (PMC4931515; doi:10.1038/srep29107)
Supplement: Supplementary Information [file srep29107-s1.pdf]

Supplementary information

Exploring Ag(111) Substrate for Epitaxially Growing Monolayer Stanene: A First-Principles Study

Junfeng Gao, Gang Zhang*[(a)], Yong-Wei Zhang*[(b)]

[(a)] E-mail: zhangg@ihpc.a-star.edu.sg; [(b)] zhangyw@ihpc.a-star.edu.sg

*Institute of High Performance Computing, A*STAR, Singapore 138632*

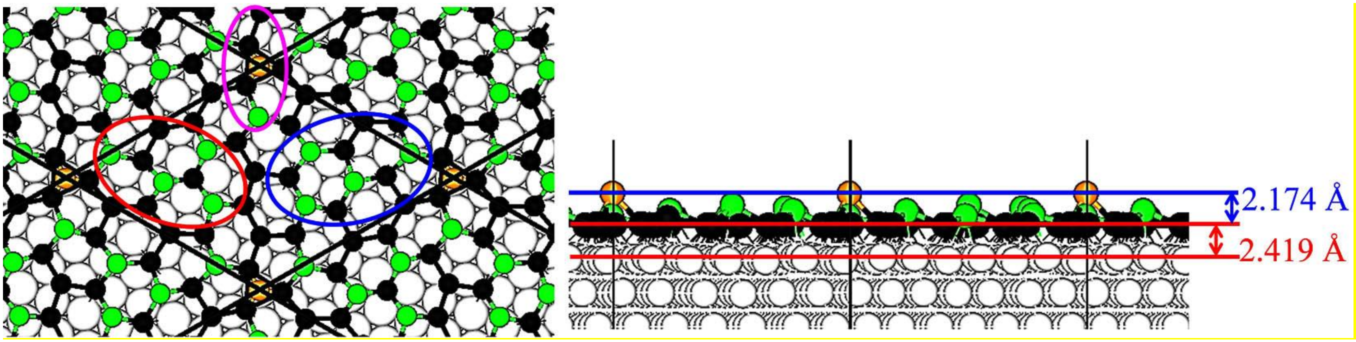

Figure S1. Optimized structure of the supercell containing a $(4\times4)R0°$ stanene on a $(\sqrt{43}\times\sqrt{43})R7.589°$ Ag(111), with dimensions of 19.229 Å × 19.229 Å × 25.000 Å. The bucking pattern of this large supercell can be recognized as a mixture of S1 (blue circle), S2 (red circle) and S3 (purple circle) structures (see Fig 1 in the main text). The binding energy between this stanene structure and Ag (111) surface is -0.058 eVÅ^{-2}, and the height of the low-level Sn atoms (black balls) to Ag(111) surface is about 2.419 Å. Both the binding energy E_b and the average height $\bar{d}$ are between those of S1(S2) and S3. The highest buckling point of this stanene structure is 2.174 Å.

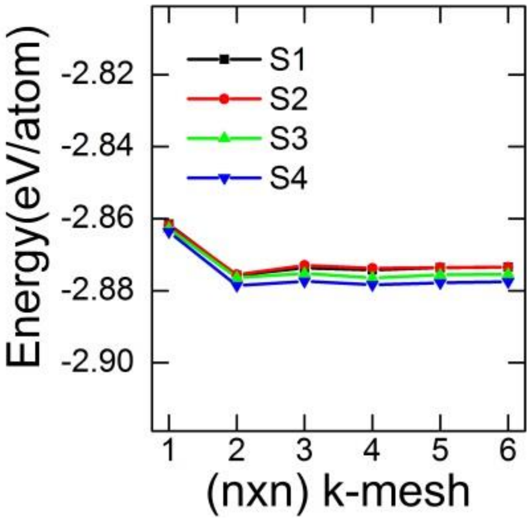

Figure S2. The K-mesh test for the structural relaxation of $(\sqrt{7}\times\sqrt{7})R19.107°$ stanene on $(\sqrt{19}\times\sqrt{19})R23.413°$ Ag(111) supercell. It is found the (2×2×1) k-mesh used in our calculations can give good energy convergence for structural relaxations; the order of stability of S1-S4 is the same as that from calculations adopted the (6×6×1) k-mesh.
